# Magneto-Electric Nano-Particles for Non-Invasive Brain Stimulation

**DOI:** 10.1371/journal.pone.0044040

**Published:** 2012-09-05

**Authors:** Kun Yue, Rakesh Guduru, Jeongmin Hong, Ping Liang, Madhavan Nair, Sakhrat Khizroev

**Affiliations:** 1 Center for Nanomedicine, College of Engineering and Computing, Florida International University, Miami, Florida, United States of America; 2 Department of Immunology, Institute of NeuroImmune Pharmacology, Herbert Wertheim College of Medicine, Florida International University, Miami, Florida, United States of America; 3 Department of Electrical Engineering, University of California Riverside, Riverside, California, United States of America; “Mario Negri” Institute for Pharmacological Research, Italy

## Abstract

This paper for the first time discusses a computational study of using magneto-electric (ME) nanoparticles to artificially stimulate the neural activity deep in the brain. The new technology provides a unique way to couple electric signals in the neural network to the magnetic dipoles in the nanoparticles with the purpose to enable a non-invasive approach. Simulations of the effect of ME nanoparticles for non-invasively stimulating the brain of a patient with Parkinson's Disease to bring the pulsed sequences of the electric field to the levels comparable to those of healthy people show that the optimized values for the concentration of the 20-nm nanoparticles (with the magneto-electric (ME) coefficient of 100 V cm^−1^ Oe^−1^ in the aqueous solution) is 3×10^6^ particles/cc, and the frequency of the externally applied 300-Oe magnetic field is 80 Hz.

## Introduction

The signaling in a biological neural network is based on a highly collective system of electric charges, neurotransmitters and action potentials. The ability to incite the neuronal charge excitations from outside with the purpose to artificially stimulate the neural network remotely (non-invasively) remains an important roadblock to enable leapfrog advances in the important area of neuroscience and related applications in medicine and neural engineering. A neural network can be considered as a complex electric circuit made of many neurons connected through synapses formed between axons and dendrites. Both types, known as chemical and electrical synapses, respectively, transfer information between adjacent axons and dendrites directly or indirectly through electric field energy. Consequently, the neural network is sensitive to external electric fields. Moreover, the ability to efficiently control the network at micro- or even nano-scale can enable unprecedented control of important brain functions. The underlying physics is still an open question because of the many technical difficulties associated with direct studies of the brain functions. The existing technology typically relies on invasive direct-contact-electrode techniques such as Deep Brain Stimulation (DBS), which is one of only a few neurosurgical methods allowed for blinded studies. Existing non-invasive brain stimulation methods include repetitive transcranial magnetic stimulation (rTMS) [1], [2] and transcranial direct current stimulation (tDCS) [3], [4]. rTMS and tDCS represent major advances of the state of the art in non-invasive brain stimulation, but the depth and locality focusing are limited in both methods [5]. In rTMS, high intensity magnetic fields are required to stimulate deep brain regions but high intensity magnetic fields may lead to undesirable side effects [6].

One potential solution to overcome the important roadblock for enabling non-invasive control of the neural network is to exploit the new concept of using magneto-electric (ME) nanoparticles we propose. ME materials represent a sub-group of multiferroic materials that are of great interest to the research community because of their ability to couple magnetic and electric fields at room temperature [7], [8]. To our knowledge, our study for the first time describes the application of ME nanoparticles to the area of brain stimulation. Particularly, our approach relies on using ME nanoparticles to achieve the following important features for non-invasive monitoring and stimulation the brain activities.

**Figure 1:**
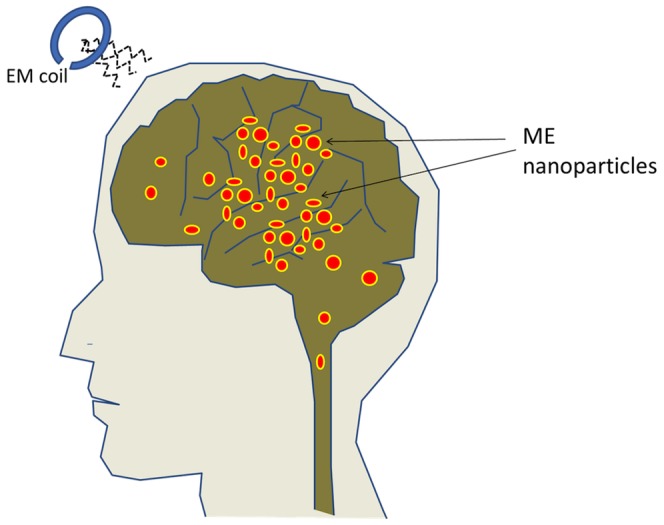
Illustration of the deep brain stimulation approach. Illustration of the deep brain stimulation approach.

First, using ME material nanoparticles through temporarily injected aqueous solutions is the key enabler for efficient coupling between magnetic and electric fields at nano- and/or micro-scale over the entire brain volume. Particularly, remotely controlled magnetic fields, instead of electric fields, can be used to induce strong local electric charge oscillations (in ME nanoparticles) that can directly interact with the neural network and therefore be used for localized and targeted brain stimulation. (See Figure 1.) Unlike surface-limited electric fields that are typically generated by invasive contact electrodes, magnetic fields generated by injected ME nanoparticles can effectively penetrate the entire brain non-invasively, and be “switched” on and off remotely using external low-energy magnetic field sources. The nanoparticles must satisfy certain requirements on the strength of magneto-electric (ME) coupling (defined by the ME coefficient).

Second, magneto-electric nanoparticles must be smaller than approximately 20 nm in diameter to penetrate the blood-brain barrier (BBB). Having the size of the ME nanoparticles within the BBB-defined boundary enables adequate delivery of the nanoparticles into selected brain regions. (There are many chemical and physical processes to synthesize ME nanoparticles with the required parameters. For instance, ion beam proximity lithography (IBPL) is a physical method that can be used to fabricate nanoparticles with a wide range of sizes, ranging from sub-10-nm to over 50 nm in diameter [9], [10].)

Third, only very low intensity external magnetic field is required to stimulate brain activity at any depth in the brain. The external magnetic field can be focused to act upon ME nanoparticles in any particular region of the brain. The external magnetic field generates AC signals in ME nanoparticles that are correlated with the frequency spectrum of the neural charge activity, which in turn causes neurons in the region to fire at similar frequencies (Figure 1). For example, provided an adequately large ME coefficient, low-energy magnetic coils can be used to trigger the required stimulation, as described below in more detail.

## Simulation

Below we describe the foundation of the modeling approach we used to model the effects of ME nanoparticles (with their externally excited electric and magnetic moments) on the brain activity. The computational procedure was built on top of a conventional model used to simulate the electric field dynamics in the neural network. For example, see the article by So *et al* to understand the underlying principles of the conventional model [11]. To account for the effect of ME nanoparticles, we made the following assumptions that can be justified at this early stage of research. First, with the average diameter of the ME nanoparticles below 20 nm in a neural system with the smallest feature size of at least an order of magnitude higher, we can use a trivial point-dipole approximation to model the electric field by each local nanoparticle [12]. Second, we assume a uniform distribution of nanoparticles in a brain region under study. The assumption is valid because the nanoparticles, due to their sub-20-nm average diameters, experience a relatively negligible resistance from the surrounding tissues and therefore their spatial distribution in the ground state can be controlled by an external magnetic field. The electric dipole moment of each nanoparticle, ***P***, is determined by the external magnetic field, **H**, according to the expression, *P_i_ = Σ_j_ α_ij_H_j_*, where *α_ij_* is the 1^st^ order linear magneto-electric (ME) tensor coefficient. Therefore, assuming an isotropic matrix (with identical diagonal coefficients and zero non-diagonal coefficients) with the typical value for *α_ii_* of 100 V cm^−1^ Oe^−1^ and a local magnetic field of 300 Oe, the polarized moment at the location of the nanoparticle would be 30 kV cm^−1^. In the current arrangement, the purpose of MF nanoparticles is to act as additional deep brain sources of electric fields that can be precisely controlled by external magnetic fields because of the non-zero ME constant. The nanoparticles can be considered as finely controlled deep brain local stimulation switches that can enable high-precision (with nanoscale localization) and high-throughput (energy-efficient) non-invasive medical procedures. To artificially trigger (stimulate) electric pulses in the brain region under study with the purpose to prevent or compensate for any illness-caused malfunctions or lapses in a periodic chain of electric signals in the parts of neural system occupied by the nanoparticles, we would apply ac magnetic fields at the matching frequencies, as described below in more detail.

## Results and Discussion

In this study, we computed the matching frequencies and concentrations of ME nanoparticles necessary to normalize the electric pulsed sequences in four regions of the brain using a model patient with Parkinson's Disease. The four regions, (i) thalamic area, (ii) subthalamic nucleus (STN), (iii) globus pallidus (GPe), and (iv) medial globus pallidus (GPi), respectively, are especially important for understanding different stages of Parkinson's Disease.

**Figure 2:**
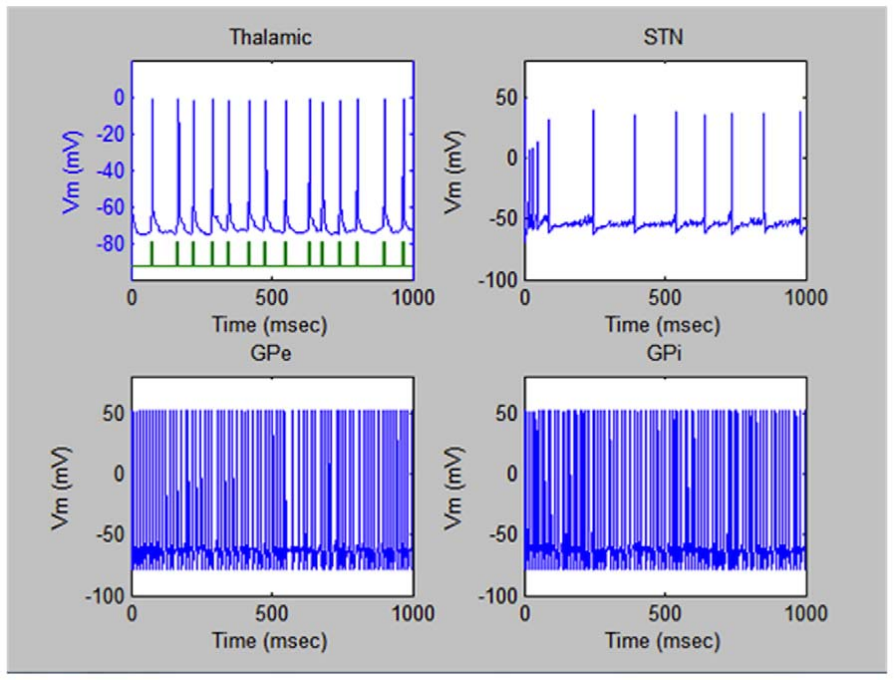
Electric pulses in the brain of a healthy person. Typical electric pulsed sequences triggered in the four regions of the brain under study of a healthy person under normal conditions.

Figure 2 illustrates typical periodic pulsed time sequences generated in the four parts of the brain of a healthy person under normal conditions. It can be noted that all the electric field pulses are quite periodic and uniform in amplitude. No lapses in the periodic sequence can be detected.

**Figure 3:**
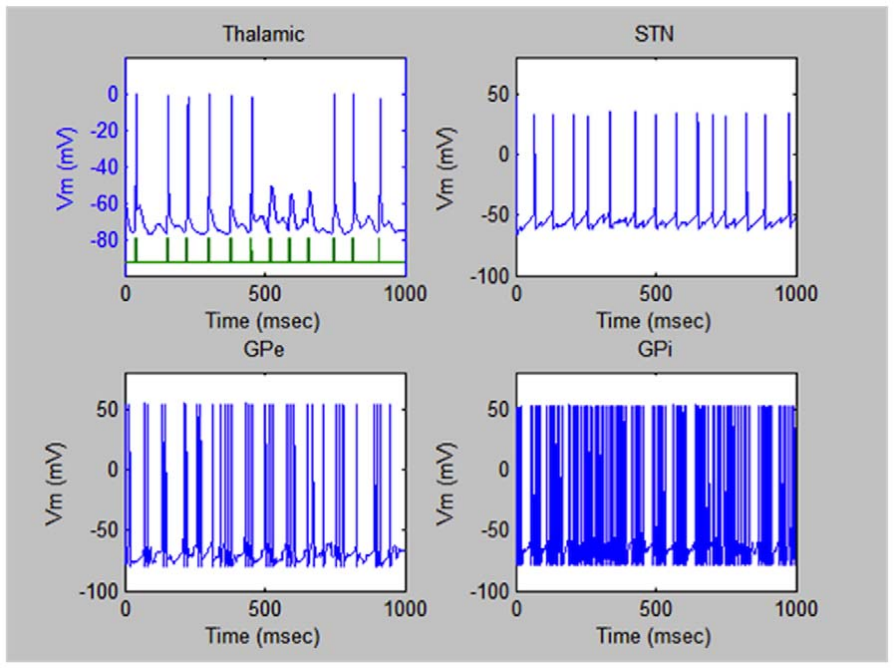
Electric pulses in the brain of a patient with Parkinson's Disease. Typical electric pulsed sequences triggered in the four regions of the brain under study of a patient suffering from Parkinson's Disease before the treatment by the ME nanoparticles. Typical electric pulsed sequences triggered in the four regions of the brain under study of a patient suffering from Parkinson's Disease before the treatment by the ME nanoparticles.

For comparison, Figure 3 illustrates typical signals in the same four parts of the brain of the patient suffering from Parkinson's Disease. The most drastic difference from the case of the healthy person is an appearance of pronounced lapses in the periodic pulsed sequences particularly in the thalamic region. Also, the periodicity of the pulses in the other regions is broken.

We studied the effects (on the potential recovery of the deteriorated electric field pulses) of the injected ME nanoparticles (in solutions) at different concentrations and stimulation frequencies (generated by a 300-Oe external AC magnetic field source). The field amplitude of 300 Oe was chosen from the requirement to maintain an energy-efficient operation while keep ME nanoparticles saturated during the procedure. The aqueous solution concentration of the nanoparticles was varied from 0 to over 10^7^ particles/cc while the frequency was varied in the range of interest, i.e., from 0 to over 1 kHz. Within the ranges of the modeling parameters, the optimum values for the nanoparticle concentration and the magnetic field excitation frequency were found to be 3×10^6^ particles/cc and 80 Hz, respectively.

**Figure 4:**
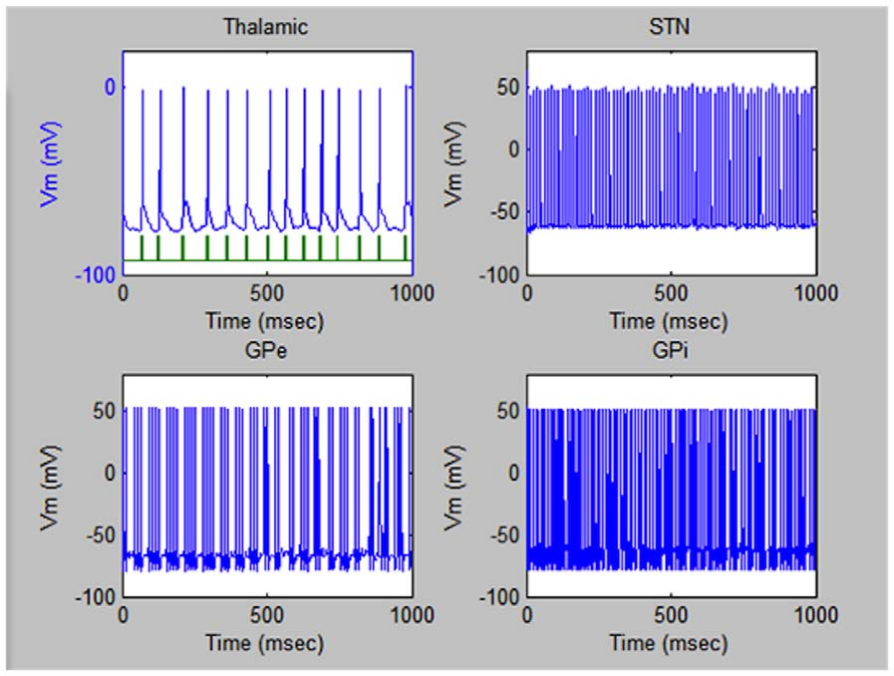
Electric pulses of a Parkinson's patient after the non-invasive ME-nanoparticle treatment. Electric pulsed sequences triggered in the four regions of the brain under study of a patient suffering from Parkinson's Disease after the treatment with the ME nanoparticles at the optimized values of the nanoparticle concentration (of 3×10 ^6^ particles/cc in aqueous solution) and the stimulation frequency (of 80 Hz).

**Figure 5:**
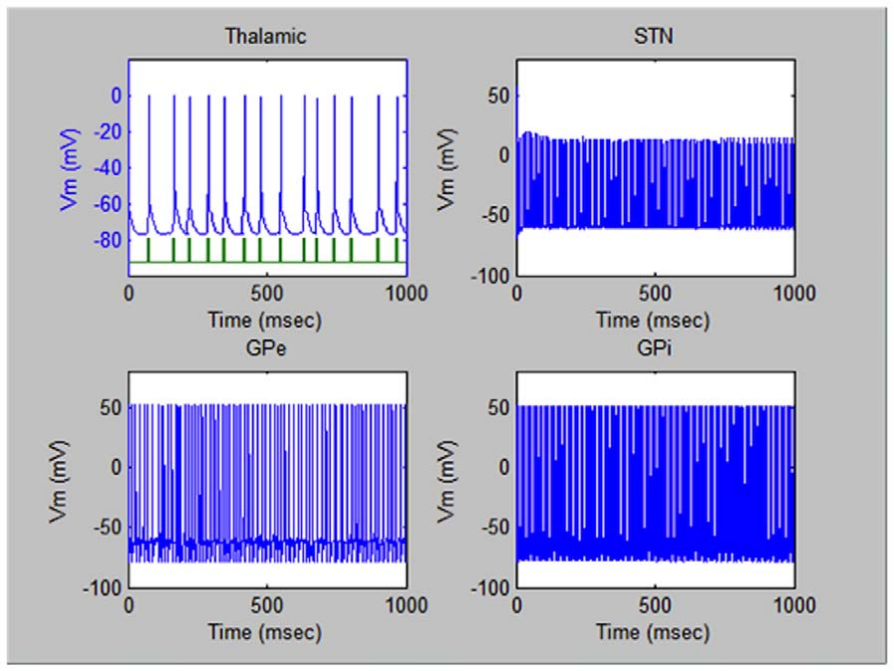
Electric pulses of a Parkinson's patient after the invasive DBS treatment. Typical electric pulsed sequences triggered in the four regions of the brain under study of a patient suffering from Parkinson's Disease after a treatment by the invasive DBS procedure with electric signals.

Figure 4 illustrates the four pulsed sequences under study as a result of the procedures with the optimized stimulation parameters. It can be observed that the most dramatically damaged signals in the Thalamic region were fully recovered during the procedure. At least partial recovery of the periodicity in the other three regions can also be observed. For comparison, the ME nanoparticle stimulation still outperformed the invasive DBS procedure with electric signal stimulation. The “recovered” signals in the four regions of the brain during the DBS procedure are shown in Figure 5. In this case, not only the periodicity was not fully recovered but also the amplitude of the signal in the STN region deteriorated compared to the normal operation.

## Conclusions

In conclusion, we modeled the effect of magneto-electric nanoparticles to non-invasively stimulate the brain of a patient with Parkinson's Disease. Using the optimized values for the concentration of the 20-nm nanoparticles (with the magneto-electric (ME) coefficient of 100 V cm^−1^ Oe^−1^ in the aqueous solution) of 3×10^6^ particles/cc and excitation frequency of the externally applied 300-Oe magnetic field of 80 Hz, the pulsed sequences of the electric field were brought to the levels comparable to those of healthy people. The preliminary results of this study suggests that using ME nanoparticles can lead the way to implementing nanotechnology to improve our understanding of the biological neural network and develop new nano-medical methods for non-invasive monitoring, preventing, and treatment of brain and other neural system diseases.
